# Bioinformatic, cell localization, and phylogenetic analyses reveal a novel family of putative lipases in *Tetrahymena thermophila*

**DOI:** 10.1093/g3journal/jkag117

**Published:** 2026-05-02

**Authors:** Jean Porterfield, Ian Knowles, Ella Wiggenhorn, Sarah Tangen, Kim Kandl

**Affiliations:** Department of Biology, St.Olaf College, Northfield, MN 55057, United States; Department of Biology, St.Olaf College, Northfield, MN 55057, United States; Department of Biology, St.Olaf College, Northfield, MN 55057, United States; Department of Biology, St.Olaf College, Northfield, MN 55057, United States; Department of Biology, St.Olaf College, Northfield, MN 55057, United States

**Keywords:** *Tetrahymena*, ciliates, lipid droplets, lipases, gene duplication

## Abstract

Lipid droplets are important and dynamic structures in all cells. Proteomic studies have shown that lipid droplets are decorated with up to hundreds of different proteins that modulate their formation, function, and interaction with other organelles. Work in our lab has shown that, like other organisms, the freshwater ciliate *Tetrahymena thermophila* increases lipid droplet formation during nutrient challenges such as starvation. Results of a *T. thermophila* proteomic screen identified lipid droplet-associated proteins, one of which (TTHERM_00784270, or 4270), is a hypothetical protein of no known function. BLAST searches of 4270 identified 15 other *T. thermophila* proteins and 19 proteins representing 6 other *Tetrahymena* species, but no orthologs outside of the genus. All of the *T. thermophila* genes are sequentially arranged along macronuclear chromosome 55, suggesting a possible gene duplication history. Phylogenetic analysis of all 35 protein sequences highlights the presence of both orthologs and paralogs in *Tetrahymena* species. Fluorescent tagging of 4 members of this family revealed that they colocalize with populations of lipid droplets. Molecular modeling as well as the presence of a pentapeptide GXSXG motif characteristic of lipases corroborate the bioinformatic evidence that we have identified a novel family of putative lipases that may play a role in how *Tetrahymena* cells store and use energy.

## Introduction

Lipid droplets are conserved lipid storage organelles found in nearly all cells. The role of lipid droplets as essential storage organelles is highlighted by human diseases associated with excessive lipid accumulation, such as obesity and fatty liver disease (Zimmermann et al. 2004; McManaman et al. 2013). Conversely, a lack of lipids or lipid droplets in adipose tissues can lead to conditions such as lipodystrophy, a diverse group of conditions characterized by loss of body fat and an increased risk of insulin resistance (Garg 2011). In addition, attention to lipid droplets has been spurred on by interests in biofuels, food production, and the recognition that they play a role in cellular responses to stress such as infection, starvation, and other nutritional challenges (Henne et al. 2018).

Lipid droplets are composed of a neutral lipid core of triacylglycerols (TAGs) and sterol esters (SEs) surrounded by a phospholipid monolayer that is decorated with associated proteins. The TAGs and SEs stored within the lipid droplets serve as efficient energy stores and as precursors for more complex molecules such as sphingolipids, signaling molecules, and steroid hormones, and lipid droplets can serve as a source of membrane that is needed for cellular and organelle membranes or autophagy (Henne et al. 2018; Lundquist et al. 2020). The size, abundance, cellular location, and function of lipid droplets are controlled by the proteins that associate with them (Bersuker and Olzmann 2017; Lundquist et al. 2020), collectively known as the lipid droplet proteome. Proteomic studies have shown that lipid droplets may be decorated with up to hundreds of different proteins, and the complement of proteins varies from one cell type to another and under different environmental conditions. Classes of validated proteins include, but are not limited to, lipid synthetic enzymes, proteins that integrate and modulate the association of lipid droplets with other organelles or aid in membrane trafficking, signaling proteins, proteins involved in degradation through the ubiquitin pathway, and lipases (Yang et al. 2012). This understanding of lipid droplet structure implies that they are not merely passive storage structures; rather, they serve as dynamic centers of intracellular activity.

Work in our lab has expanded the study of lipid droplets and their associated proteins to include the single-celled ciliate *Tetrahymena thermophila*, a model organism that is evolutionarily distant from those organisms in which lipid droplets have been primarily studied (ie animals, yeast, and plants). With respect to lipids, *Tetrahymena* have both shared and unusual characteristics compared to other eukaryotes. Like other organisms, *Tetrahymena* accumulate lipid droplets in response to nutrient deprivation, including starvation and stationary phase growth (Listenberger et al. 2025). Triacylglycerides are the main storage lipids, and *Tetrahymena* have components of the pathways that generate triacylglycerols via de novo synthesis in the ER membrane including GPAT, AGPAT, PAH/lipin, and DGAT (Pillai et al. 2017). *Tetrahymena* is like other cells in that its membranes are composed primarily of phosphatidylethanolamine, phosphatidylcholine, and sterol (Nozawa and Thompson 1971). However, they have a unique sterol (called tetrahymenol), and many of their phospholipids include ether-linked acyl chains rather than ester linkages (Thompson 1967; Levy and Elliott 1968; Nozawa and Thompson 1972; Raugi et al. 1973). In addition, *Tetrahymena* do not appear to have homologs of canonical lipid droplet-associated proteins, such as members of the perilipin family that are found in all eukaryotes except plant lineages (Lundquist et al. 2020).

One of the protein categories commonly identified in lipid droplet proteomic screens is lipases, enzymes that hydrolyze triacylglycerols. Ciliate lipases have been isolated from extracellular media (Florin-Christensen et al. 1986; Brock et al. 2016), and some have been patented for use as a therapy replacement of human pancreatic enzymes (Brock et al. 2016). One characteristic of many lipases is a conserved GXSXG motif associated with catalytic activity (Derewenda and Derewenda 1991; Ollis et al. 1992). Like many proteins, lipases are often encoded by genes in gene families, sometimes in the form of paralogs resulting from tandem duplication events. For example, the different lipase family members in the fungal species *Malassezia restricta* may contribute to its disease pathogenesis (Park et al. 2021). In addition, members of the soybean GDSL-type esterase/lipase protein (GELPs) gene family are distributed among chromosomes, with several locations showing tandemly duplicated genes (Su et al. 2020), and the pancreatic lipase genes in mammals comprise 3 tandem paralogs (Tang et al. 2022). Gene families of paralogs are common in ciliates, such as the cluster of tandem paralogs within dynamins (Chalker 2008), tubulins (Su et al. 2024), and the histone H4 gene family (Katz et al. 2004). Gene duplication and gene families have played important roles in the evolution of many taxa (Demuth and Hahn 2009), including ciliates (Gao et al. 2014). In *Tetrahymena*, most of the tandemly duplicated gene families appear to be localized instead of being attributed to whole-genome duplications (Eisen et al. 2006). No matter their origins, paralogous genes often play important roles in the diversity of proteins and their functions in cells (Deem and Brisson 2024).

Here we use a variety of methods to characterize a protein, TTHERM_00784270 (“4270”), that we identified through a screen for lipid droplet-associated proteins. BLAST searches and subsequent bioinformatic analyses revealed that 4270 is one of 16 putative paralogs in the *T. thermophila* genome, arranged in tandem on macronuclear chromosome 55. Orthologs were identified in 8 other species of *Tetrahymena*, but not in any other taxa. Most of the homologs contain a canonical GHSLG lipase motif, and molecular modeling suggests these proteins could be TAG lipases. Consistent with this observation, fluorescent tagging of 4270 and 3 additional family members confirmed their association with lipid droplets.

## Materials and methods

### Proteomic screen

Lipid droplets were isolated from starved *Tetrahymena* cells, when lipid droplets are abundant, by flotation after sucrose density gradient ultracentrifugation. Lipid droplet SDS-PAGE bands were excised, reduced, alkylated, and digested overnight using sequencing grade trypsin (Cole et al. 2008). The resulting peptide mixtures from each band were analyzed on a MALDI-TOF/TOF instrument in positive-ion MS mode using α-cyano-4-hydroxycinnamic acid matrix. The results were screened against a database of known and predicted *Tetrahymena* proteins using the Mascot search engine software. MOWSE scores (Pappin et al. 1993) were used to determine protein identifications, with a lower MOWSE score of 30 used as the cutoff for an initial positive identification. At least one peptide from each band/digest was further analyzed by MS/MS to ensure sequence confirmation with the MS-identified protein. This resulted in 21 proteins identified.

### Bioinformatics

One of the *Tetrahymena* hypothetical proteins (4270) identified in the proteomic screen was used in searches performed using the BLAST+ suite (Camacho et al. 2009) at the National Center for Biotechnology Information (NCBI). The amino acid sequence for 4270, as listed on its *Tetrahymena* Genome Database (TGD) page at tet.ciliate.org (Stover et al. 2012), was searched against the NCBI's nonredundant protein sequences database using the BLASTp (protein–protein BLAST) algorithm. A resource called the *Tetrahymena* Comparative Genome Database (TCGD; Yang et al. 2019) contains 8 *Tetrahymena* genomes that are not found in GenBank (Sayers et al. 2025), so protein sequence 4270 was queried in a BLASTp within TCGD. In order to identify sequences that might have functional similarities despite being evolutionarily distant, a DELTA-BLAST (Domain Enhanced Lookup Time Accelerated BLAST; Boratyn et al. 2012) of sequence 4270 was performed. In addition, protein sequence 4270 was used to query several protein structure databases (AlphaFold; Varadi et al. 2024, Protein Data Bank; Berman et al. 2000, and Swiss-Prot; Bateman et al. 2025) in a profile hidden Markov model sequence similarity search (PHMMER) with a substitution scoring matrix (BLOSUM45) best for divergent sequences, employed from the European Molecular Biology Laboratory's European Bioinformatics Institute (EMBL-EBI) platform (Madeira et al. 2024). Gene expression data for the *T. thermophila* sequences were gathered in TGD from 5 transcriptome data tracks, and from microarray analysis results (Miao et al. 2009) presented on each gene's individual TGD page.

### Phylogenetic analysis

All of the putatively homologous protein sequences were obtained from TGD (for *T. thermophila*) or TCGD (for all other *Tetrahymena* species) and were aligned using the MUSCLE algorithm within SnapGene (version 8.0.3, GSL Biotech, LLC, San Diego, California). Because several taxa contained long stretches of amino acids that no other sequences had, and there was low alignability in many areas of sequence, the alignment was trimmed by hand to include the longest stretch of contiguous conserved amino acid positions (maximum length 374). This trimmed alignment was imported into MEGA12 (Kumar et al. 2024), wherein a model selection analysis determined that the data best fit the WAG (Whelan and Goldman 2001) base substitution model allowing among-site rate variation and invariant sites, and using amino acid frequencies assessed from the data set. This model was used in a maximum likelihood analysis, where the initial tree had the lowest log likelihood in both neighbor-joining and maximum parsimony analyses. Support for the branches in the tree was tested with adaptive bootstrapping using a minimum replicate number of 25 and a standard error of 0.05.

### Construction of tagged proteins and selection of transformed *Tetrahymena*

Oligonucleotide primers recognizing the protein-coding sequences for genes 4270, 4260, 4250, and 2110 were designed and used in high-fidelity PCR using either Phusion or Q5 DNA Polymerase (New England Biolabs). Primer pairs for N-terminal tagging recognized the first codon after the ATG and ended after the TGA stop codon. Primer pairs for C-terminal tagging recognized 6 nucleotides upstream of the ATG start codon and ended just before the TGA stop codon. Resulting amplicons were directionally TOPO cloned into the pENTR/D-TOPO entry vector (Thermo Fisher Scientific) using CACC/GTGG that was added to the 5′ end of each oligonucleotide. The pENTR clone was recombined using the LR Clonase (Thermo Fisher Scientific) reaction into the Gateway-based *T. thermophila* destination expression vectors, pBS-mCherry-gtw (N-terminal tagging) and pBS-YFP-gtw (C-terminal tagging), a gift from Doug Chalker (Malone et al. 2008). Both plasmids contain an *MTT1*-inducible promoter, and a cycloheximide-resistant *Tetrahymena* rpl29 allele to allow integration by homologous recombination into the endogenous *RPL29* locus. Plasmids were confirmed by whole plasmid sequencing (Eurofins Genomics). Prior to biolistic transformation, plasmid constructs were linearized with HindIII or SacI/PvuI and transformed into *Tetrahymena* cells starved for 18 h in 10 mM Tris, pH 7.5. Transformed cells were selected in Neff medium (0.25% proteose peptone, 0.25% yeast extract, 0.5% dextrose, 33.3 μM FeCl_3_) containing 12.5 μg/ml cycloheximide. After initial selection at 12.5 μg/ml cycloheximide, the concentration was gradually increased to 50 μg/ml cycloheximide. This more stringent selection ensures that transformation was successful and increases the copy number of the gene for the tagged protein by phenotypic assortment. Transformants were further verified by fluorescence microscopy with or without cadmium induction.

### Cell growth

Wild-type CU428.2 strain of *T. thermophila* (*Tetrahymena* Stock Center at Washington University) biolistically transformed with YFP- or mCherry-tagged constructs were grown at 30 °C in modified Neff with 25 μg/ml cycloheximide to log phase, ie between 2 × 10^5^ and 5 × 10^5^ cells/ml. To induce expression of the tagged proteins, 0.5 μg/ml CdCl_2_ was added to cell cultures for 17 h, while maintaining cells in log phase. For starvation, cells in log phase were washed twice and resuspended in 10 mM Tris, pH 7.5, at a density of approximately 3 × 10^5^ cells/ml.

### Fluorescent microscopy

Expression of tagged proteins was visualized following induction with 0.5 μg/ml CdCl_2_for 17 h and starvation for 6 h. Lipid droplets were visualized following 30 min incubation with 1 μg/ml BODIPY 493/503 (Thermo Fisher Scientific) or 2 μM monodansylpentane (MDH, Abcepta). Live cells were examined using the 40× objective on an Olympus BX53 microscope equipped with a FITC or TRITC filter and an ORCA-Flash 4.0LT+ camera. Single-plane images were acquired sequentially, merged, and pseudocolored using CellSens software (Evident Scientific) and exported for analysis and processing as TIFF files.

### Image processing and analysis

For analysis of co-occurrence between fluorescent-tagged proteins and lipid droplets, TIFF images were opened in ImageJ (Schneider et al. 2012) and cropped tightly around individual cells, and background was subtracted using a rolling ball radius of 12 pixels, a value larger than the puncta staining consistent with lipid droplets. The ImageJ JACoP plugin (Bolte and Cordelieres 2006) was used to determine Pearson's correlation coefficient with Costes’ randomization and Manders’ split coefficient using the JACoP default threshold parameters. Data were graphed and analyzed using Prism 10 (GraphPad Software, Inc.).

### Molecular modeling

Molecular models were generated using Chai Discovery Version 1 (Chai Discovery et al. 2024), predicted without MSAs or templates. Full-length sequences of proteins, predicted from the sequenced genome, were from TGD (Stover et al. 2012). Each protein was modeled interacting with a triacylglycerol (1,2-dioleoyl-3-palmitoylglycerol) ligand, generated using a SMILES Id from PubChem (CCCCCCCCCCCCCCCC(=O)OCC(COC(=O)CCCCCCC/C=C\CCCCCCCC)OC(=O)CCCCCCC/C=C\CCCCCCCC). Confidence in the structures and the protein/ligand interactions was determined with predicted template modeling (pTM) and interface predicted template modeling (ipTM) scores. Models were observed and imaged using UCSF ChimeraX version 1.10 (Pettersen et al. 2004). ChimeraX was used to measure the distance from the center of the GXSXG motif to the closest point of the ligand.

## Results and discussion

### Bioinformatic characterization of a family of *Tetrahymena*-specific genes

To better understand fat storage and utilization in *T. thermophila*, we performed a preliminary proteomic screen for lipid droplet-associated proteins by isolation of lipid droplets during starvation, when lipid droplets accumulate (Listenberger et al. 2025). This screen identified 21 proteins, including TTHERM_00784270, or “4270.”

The protein–protein BLAST (blastp) of the 4270 amino acid sequence against the NCBI nonredundant protein sequences database identified its own accession, 14 other *T. thermophila* proteins, and 6 proteins in 2 other *Tetrahymena* species (*T. malaccensis* and *T. utriculariae*), all with *E*-values of 1e-26 or less (Supplementary Table 1). Using the DELTA-BLAST algorithm resulted in hits within the same 21 *Tetrahymena* sequences with E-values of 5e-25 or lower, followed by a list of bacterial chromosomal segregation proteins from the genera *Bacillota*, *Bacillus*, *Chlorobaculum*, and *Lactobacillus* with E-values of 1e-17 or higher. No other taxa were represented in the first 500 sequence matches (representing 1,402 total hits). The profile hidden Markov model sequence similarity search (PHMMER) resulted in no hits when queried against PDB and Swiss-Prot, and 15 hits when queried against AlphaFold. The 15 AlphaFold hits included no new sequences beyond the *T. thermophila* genes already in the analysis. These data suggest that this gene family is unique to *Tetrahymena*.

Results of querying 4270 with BLASTp in the *Tetrahymena* Comparative Genome Database (TCGD) included, with E-values of 1.18e-9 or lower, hits with 13 sequences from 6 additional *Tetrahymena* species: 1 sequence from each of *T. empidokyrea* and *T. vorax*; 2 sequences from each of *T. borealis*, *T. canadensis*, and *T. shanghaiensis*; 5 sequences from *T. pyriformis*; and the same *T. malaccensis* and *T. thermophila* hits as with the GenBank BLASTp searches (Supplementary Table 1). No matches were found with the other 2 *Tetrahymena* species in TCGD (*T. elliotti* and *T. paravorax*). All of the proteins associated with these sequences either have the ontology of an alpha–beta hydrolase fold or transmembrane protein or are designated as uncharacterized or hypothetical (Supplementary Table 1). Since lipases are a group of alpha–beta hydrolase fold enzymes and can be transmembrane (eg Lazniewski et al. 2011), the protein ontologies are consistent with the *Tetrahymena* proteins being lipases, especially when viewed in light of the presence of a GXSXG lipase motif in most sequences (further discussed later).

**Fig. 1.**
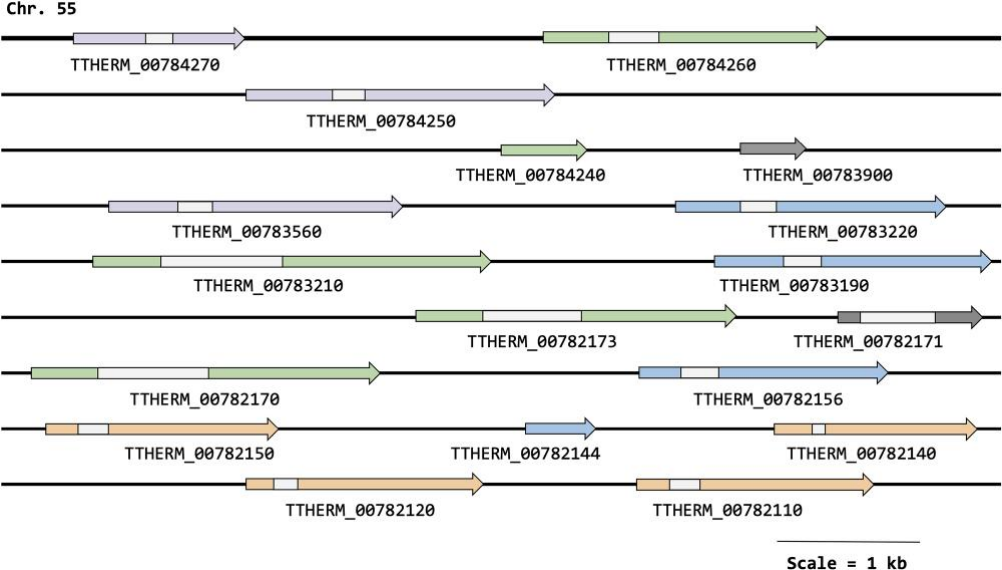
Arrangement of TTHERM_00784270 (“4270”) and putative paralogs. JBrowse software (Skinner et al. 2009) within TGD was used to obtain the *T. thermophila* gene sequences and chromosomal locations 781,402 (start codon of 4270) to position 843,474 (stop codon of 2110) on *T. thermophila* chromosome 55 which are depicted to scale. Darker boxes indicate exons, and lighter boxes indicate introns; 14 of the 18 genes in this region are composed of 2 exons, while 4 (4240, 3900, 2171, 2144) have no introns. Colors represent the clade that includes that gene's protein sequence (see phylogeny in Fig. 2a), while gray represents the 2 genes in this region for which homology with the rest of the sequences could not be established and were thus not included in phylogenetic analysis.

While we identified 15 *T. thermophila* genes through a BLASTp search at NCBI, the same BLASTp search at the *Tetrahymena* Genome Database (TGD) and use of JBrowse software (Skinner et al. 2009) revealed that these 15, plus 3 additional genes, are all arranged on the same coding strand near the end of macronuclear chromosome 55 (Fig. 1). The 3 additional genes are present in TGD but not in NCBI because they were identified in the most updated annotation of TGD (Fig. 1 and Supplementary Table 1).

### Phylogenetic analyses

Information from alignments guided decisions about which of the 18 *T. thermophila* genes within the region on chromosome 55 (Fig. 1) should be included in phylogenetic analysis. With a much smaller size, genes 4240, 3900, 2171, and 2144 are dissimilar from the other 14 genes in the array. Multiple sequence alignment using the MUSCLE algorithm in SnapGene (GSL Biotech, LLC) revealed that 3900 and 2171 aligned sporadically with the other 16 *T. thermophila* sequences (and with each other), and did not include any version of the aforementioned GXSXG lipase motif that almost every other *Tetrahymena* sequence in the dataset had. However, 4240 and 2144 aligned well with a region associated with the last exon of the 14 multiple-exon sequences, including the conserved GXSXG motif. Thus, genes 3900 and 2171 were excluded, leaving 16 *T. thermophila* protein sequences to include in phylogenetic analysis.

**Fig. 2.**
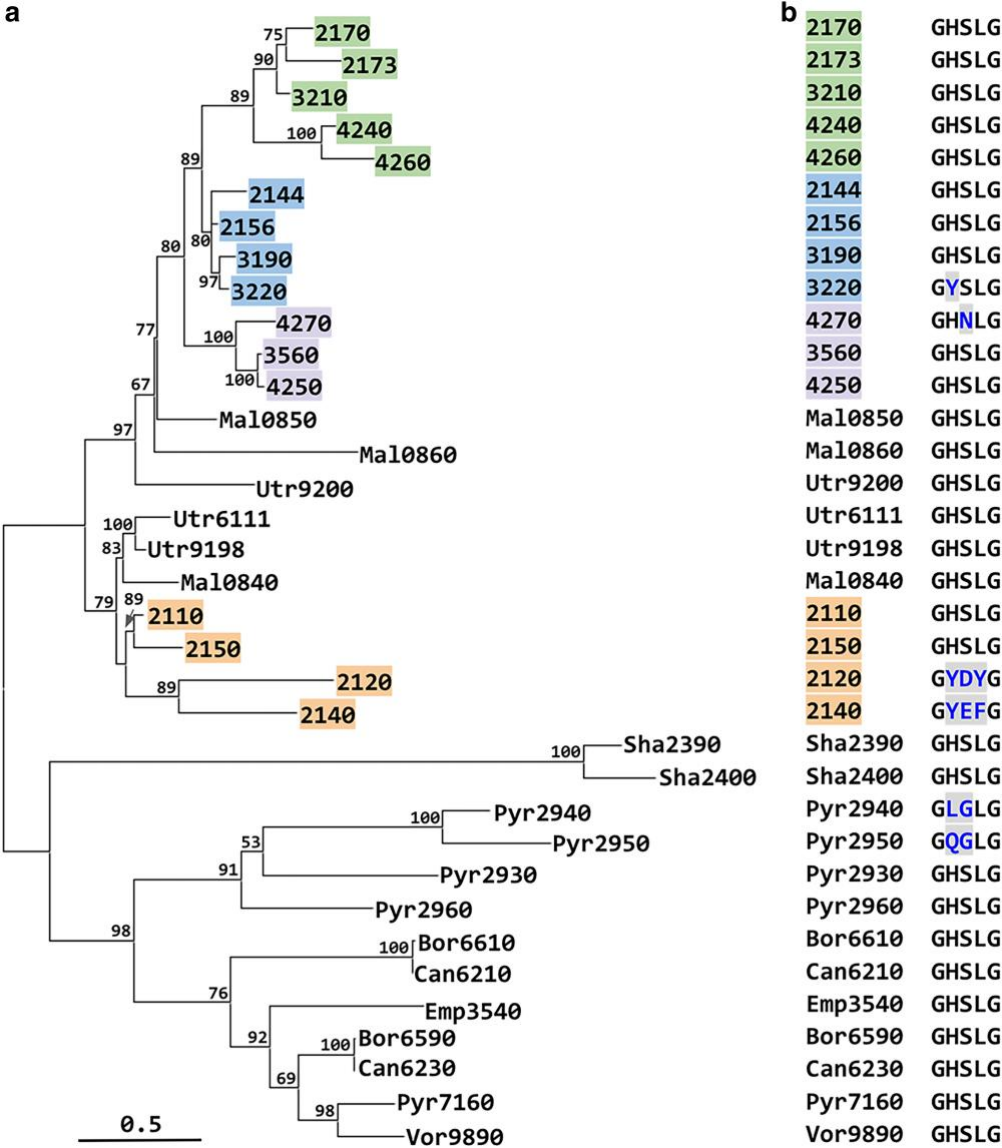
Phylogenetic structure and variation in the lipase motif among sequences. a) Maximum likelihood phylogeny of all 35 putatively homologous protein sequences, rooted at the common ancestor of the *T malaccensis*, *T. thermophila*, and *T. utriculariae* clade, and the clade containing the rest of the species in the analysis. Branch support values on the tree represent the percentage of trees out of 107 bootstrapping replicates (determined adaptively; Kumar et al. 2024) that contain that branch structure. The scale bar represents amino acid substitutions per site. b) Alignment of the lipase motif in the 35 amino acid sequences; the flanking glycines are invariant, and most of the sequences contain a middle serine flanked by histidines and leucines.

The 16 *T. thermophila* sequences were aligned with the 19 sequences from the 8 other *Tetrahymena* species identified in NCBI or TCGD, resulting in a data set of 35 amino acid sequences for phylogenetic analysis. As determined in MEGA12 (Kumar et al. 2024), the model that best fit the hand-trimmed protein sequence alignment was the WAG (Whelan and Goldman 2001) amino acid substitution model, incorporating among-site rate variation (modeled with a gamma distribution across 4 rate categories), accounting for invariant sites (2.62% were invariant), and using amino acid frequencies estimated from the data. The phylogeny generated with this model is shown in Fig. 2a. The root of this tree was determined by comparing the relationships among taxa in this tree with the relationships of the same taxa in published phylogenies of *Tetrahymena* based on cytochrome oxidase I sequences (Chantangsi and Lynn 2008, Rataj and Vďačný 2020). In those studies, a clade containing *T. malaccensis*, *T. thermophila*, and *T. utriculariae* is the sister group to a clade that contains the rest of the *Tetrahymena* species that are represented in the current study. Branch support values on the tree represent the percentage of trees out of 107 bootstrapping replicates (determined adaptively; Kumar et al. 2024) that contain that branch structure.

Overall, the phylogeny suggests that both speciation (resulting in orthologs) and gene duplication events (resulting in paralogs) have influenced the evolution of these sequences in *Tetrahymena*. There appear to be 2 groups of paralogs among the 16 *T. thermophila* sequences: 1 clade consisting of 12 sequences sister to orthologous sequence 0850 from *T. malaccensis* (Mal0850) and 1 clade consisting of 4 sequences (2110, 2120, 2140, 2150) with a sister clade of 2 *T. utriculariae* and 1 *T. malaccensis* sequence. While some gene duplications have given rise to paralogs in species other than *T. thermophila* (eg 4 of the 5 *T. pyriformis* “Pyr” sequences, the 2 *T. shanghaiensis* “Sha” sequences, and 2 of the *T. utriculariae* “Utr” sequences), there has been more gene duplication within *T. thermophila* than in any other represented *Tetrahymena* species for this new gene family. This is consistent with findings that *T. thermophila* genomes often contain duplicated copies of genes, especially those that encode enzymes involved in resource specialization (Eisen et al. 2006). As more *Tetrahymena* species’ sequences become available, further phylogenetic analysis, ideally coupled with synteny analysis, could provide more insight into the evolution of this family of proteins within the genus.

Comparing the *T. thermophila* sequences in the phylogeny (Fig. 2a) with their gene loci (Fig. 1) shows that, with the exception of 2 pairs of genes (2120/2140 and 2173/2170), no gene is chromosomally adjacent to its phylogenetic sister sequence. For example, the “green” sequences 2170, 2173, 3210, 4240, and 4260 form a clade that is closely related to a “blue” clade of 2144, 2156, 3190, and 3220, and the combined “blue” plus “green” clade is sister to a “purple” clade of sequences 4270, 4250, and 3560. However, the order of these 12 genes on the chromosome is 4270 (purple), 4260 (green), 4250 (purple), 4240 (green), 3560 (purple), 3220 (blue), 3210 (green), 3190 (blue), 2173 (green), 2170 (green), and 2156 (blue). This is consistent with segmented duplication events (eg Birchler and Yang 2022); however, not all of these duplications need to be an evolutionary event. *T. thermophila*, like many other ciliates, is notorious for gene rearrangements when forming its macronucleus (from which genes are expressed in the cell) from its micronucleus (which contains a complete set of genes) after conjugation (Hamilton et al. 2016; Bétermier et al. 2023).

The specific amino acid sequence at the GXSXG lipase motif for each taxon is shown in Fig. 2b; 29 of the 35 protein sequences specifically contain a GHSLG, with invariant flanking glycines. These small, neutral glycine residues are thought to facilitate the catalytic activity of the serine residue by flexibly allowing the serine to be at the point of the nucleophilic elbow in proximity to a substrate (Carr and Ollis 2009). With respect to the middle positions of the pentapeptide, there are 6 non-GHSLG sequences: 4270 (asparagine instead of serine), 3220 (tyrosine instead of histidine), 2120 (aspartic acid instead of serine, tyrosines instead of the histidine and leucine), 2140 (glutamic acid instead of serine, tyrosine instead of histidine, phenylalanine instead of leucine), Pyr2940 (glycine instead of serine, leucine instead of histidine), and Pyr2950 (glycine instead of serine, glutamine instead of histidine). In most cases, mutation of an amino acid in a highly conserved functional motif would be associated with either pseudogenization or refunctionalization (eg Lynch and Conery 2000); one way to support the latter is to find evidence that the duplicate genes are expressed.

Of the 12 *T. thermophila* genes for which there are microarray data at TGD (Miao et al. 2009), most have an expression peak within the first 4 h of starvation, and most show another peak of expression within the first 6 h of conjugation (sexual reproduction; Supplementary Fig. 1). TGD also presents RNAseq data tracks from M. Cervantes (unpubl.) and Ye et al. (2025); data from 5 of these tracks are shown in Supplementary Fig. 2. These 5 RNAseq data tracks observed with JBrowse in TGD yielded the following assessment: 3 genes (4270, 4260, 2173) showed highly similar expression patterns across all 5 tracks; 5 genes (4250, 2150, 2140, 2120, 2110) showed more expression in the log-phase tracks than in the starvation or conjugation tracks; 2 genes (3560, 2170) showed low and variable expression across all tracks; 1 gene (2156) showed low levels of expression only in the starvation and conjugation tracks; 2 genes showed low expression in just 1 track (3220 in the starvation 1 h track and 3190 in a log-phase track); and 3 genes (4240, 3210, 2144) did not show expression in any of the tracks (Supplementary Fig. 2). Of the 4 non-GHSLG *T. thermophila* sequences for which there are transcriptomic data tracks (Supplementary Fig. 2), all exhibit significant expression in all 5 tracks except for 3220, which shows low expression levels in the 2 starvation condition tracks. Overall, the *T. thermophila* proteins lacking a GHSLG lipase motif still seem to be functional rather than pseudogenized, at least based on available transcriptomic data.

### Localization of proteins at lipid droplets

**Fig. 3.**
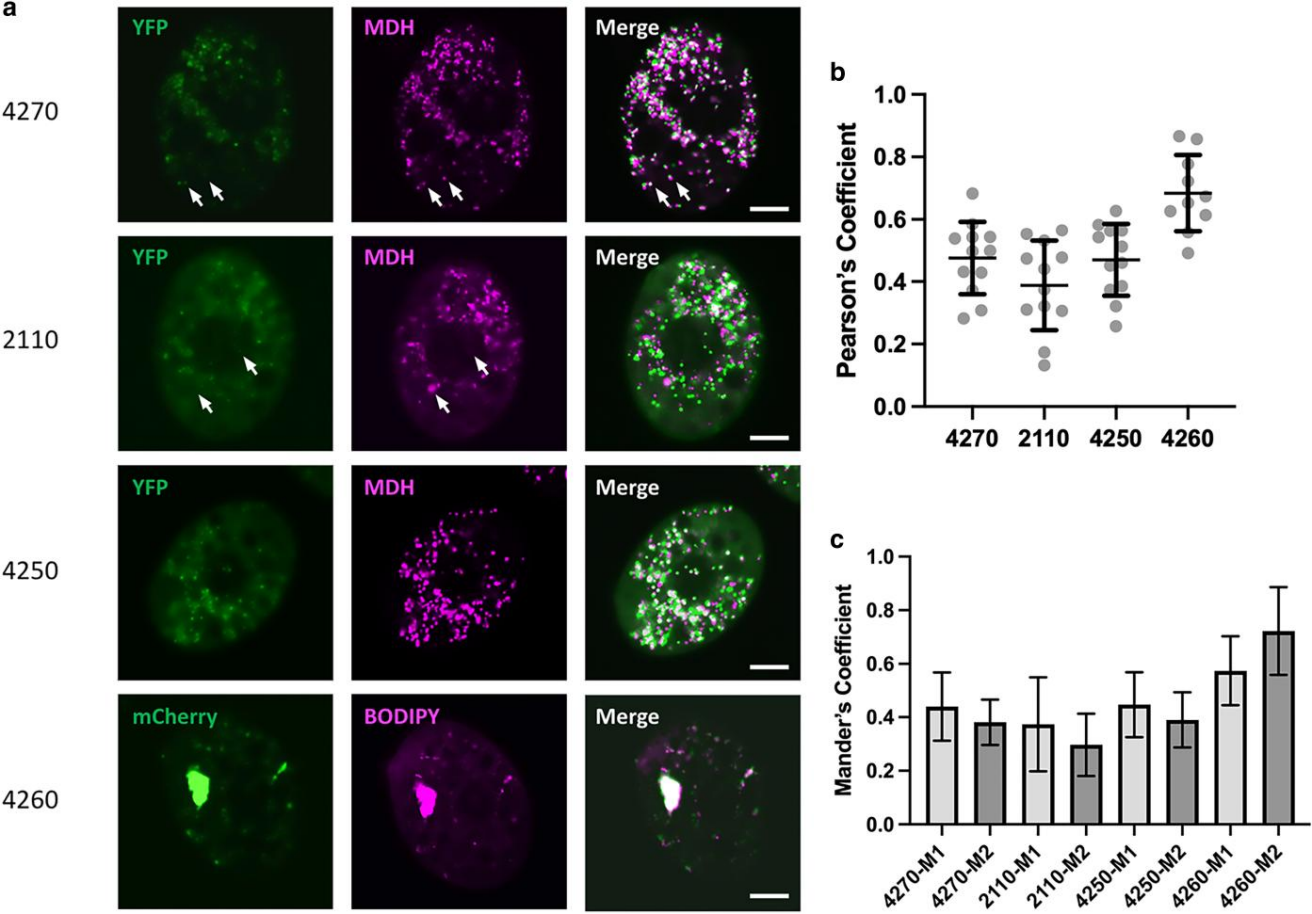
4270 and paralogs 2110, 4250, and 4260 colocalize with lipid droplets in *Tetrahymena*. a) Cells expressing yellow fluorescent protein (YFP)-tagged 4270, 2110, and 4250 or mCherry-tagged 4260 were grown in rich Neff Media to log phase under selective conditions (25 μg/ml cycloheximide) with cadmium induction in 0.5 μg/ml CdCl_2_. Cells were washed in 10 mM Tris, pH 7.5, and starved for 6 h. Lipid droplets were visualized after 30 min of incubation with the lipid droplet stain MDH (Abcepta) or BODIPY 493/503 (Invitrogen). Scale bar, 20 µm. Quantification of the colocalization and correlation between the tagged protein and lipid droplet signals was performed using the JACoP plugin for ImageJ. Pearson's correlation coefficient (b) and Manders’ split coefficient (c) are shown. For each experimental group, *n* ≥ 10.

To validate 4270 and 3 of its paralogs as lipid droplet-associated proteins, we examined the co-occurrence of their tagged versions with dyed lipid droplets (Fig. 3a). While not all of the structures stained with the fluorescently tagged proteins align perfectly with the lipid droplet stains MDH or BODIPY 493/503, the staining patterns were generally similar. Lack of co-occurrence in the third (merge) column may be due to shifting of live cells during sequential imaging with different fluorescence filters. For example, in 4270 (Fig. 3a, top row), the YFP staining indicated by the left arrow is in the same position as the MDH staining and yields a white merged color, indicating that 4270 associates with lipid droplets; however, the right arrow shows what appears to be a shift in position of the 4270 protein and lipid droplets relative to each other during live cell imaging.

Pearson's correlation coefficient is a measure of the correlation between the 2 fluorescent channels with values ranging from +1 (perfectly correlated) to −1 (perfectly anticorrelated). Because the imaging and analysis in this study were performed on live cells, a Pearson's correlation coefficient of 1 is not expected. Figure 3b depicts an average Pearson's correlation coefficient of 0.388 (for 2110) to 0.684 (for 4260). Another method of assessing potential artifacts of live cell imaging is Costes’ randomization, in which the pixels from one of the channels are randomized; when applied to these images, this method yielded Pearson's coefficients of approximately 0 (ie 0.0 ± 0.008 to 0.0 ± 0.004, data not shown).

It is likely that there are different lipid droplet and protein populations within cells, and not all lipid droplet populations are decorated with these proteins under the condition examined (starved for 6 h). For example, in 2110 (Fig. 3a), the left arrow indicates a structure that is stained by the lipid dye MDH, but there is no corresponding staining in the YFP channel, and the right arrow indicates the opposite situation with a YFP-stained structure that has no counterpart in the MDH channel. To further study the overlap between the tagged proteins and lipid droplets, we determined the Manders’ split coefficients to assess the co-occurrence of lipid droplet signal with tagged proteins (M1, Fig. 3c) and the tagged protein with lipid droplets (M2, Fig. 3c). Manders’ coefficients range from 0 to 1, with 1 reflecting perfect co-occurrence of the pixel signal in both channels. The Manders’ coefficients for these images ranged from 0.297 (M2 for 2110) to 0.723 (M2 for 4260). One-way ANOVA comparison tests for the M1 and M2 coefficients for 4270, 2110, and 4250 were not significant; the M1 and M2 coefficients for 4260 were weakly significant (*P* = 0.0476) suggesting that more of the 4260 protein colocalizes with lipid droplets than vice versa. However, in the case of 4260, the tagging method used may have created microscopy artifacts that led to a clumping of lipid droplets (Fig. 3a, bottom row). Such clumps could result from protein aggregation caused by steric hindrance from the N-terminal mCherry tag (236 amino acids and a predicted molecular mass of 28 kDa) and/or by the overexpression levels of 4260 from its inducible promoter. In fact, tagging 4260 at the C-terminus with YFP (238 amino acids) caused similar clumping (not shown), indicating that the position of the tag relative to the protein does not affect clustering in 4260 stained cells.

### Molecular modeling of structures and interaction with triacylglycerol

**Fig. 4.**
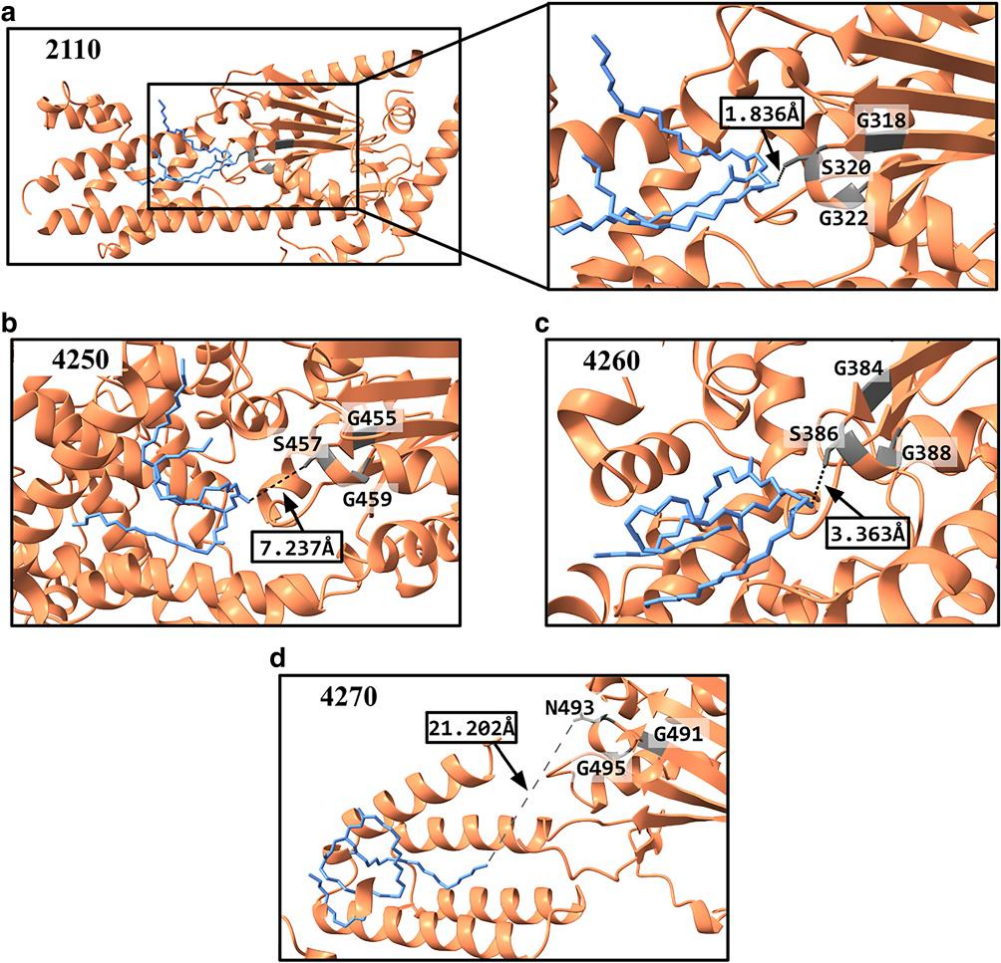
Positioning of a triglyceride relative to the lipase motif in Chai Discovery structural models of proteins 2110, 4250, 4260, and 4270. a) Model of 2110 with inset zoomed in on the area containing the triacylglycerol and the GXSXG motif, indicating the distance in angstroms from the center of the motif to the closest point of the triacylglycerol. Zoomed-in active sites of protein 4250 (b) and 4260 (c) show a configuration similar to 2110 (inset a), while the 4270 model (d) shows that an asparagine residue (N493) replaces the canonical serine in the motif; the closest point of the triacylglycerol is farther (21.202 Å) from the center of the motif.

Our microscopy data show an association between tagged proteins and lipid droplet stains. This observation, alongside comparisons of the GXSXG motif (Fig. 2b), provides evidence for lipase categorization for some of these proteins, an idea we further explored through molecular modeling. Molecular structure predictions with Chai Discovery (Chai Discovery et al. 2024) of the 4 proteins that were tagged, along with a triacylglycerol (1,2-dioleoyl-3-palmitoylglycerol), are shown in Fig. 4. The predicted structure of 2110 (Fig. 4a) shows the triacylglycerol placed adjacent to the GXSXG lipase motif (Fig. 4a, inset). The modeled triacylglycerol–protein interactions are shown for the other 3 proteins (4250, 4260, 4270) in Fig. 4b to d.

Three of the 4 tagged proteins (2110, 4250, 4260) contain the canonical GHSLG motif associated with lipases and other alpha–beta hydrolase enzymes (Holm et al. 1994); the models of these 3 proteins with a triacylglycerol show the ligand near the serine of the nucleophilic elbow of the GXSXG motif (Fig. 4a to c). Protein 4270 has an asparagine instead of a serine at this position, and as modeled, the triacylglycerol is positioned more than 21Å from the motif (Fig. 4d). While this longer distance suggests that protein 4270 may not function similarly to the other 3 proteins modeled, it is highly expressed in diverse conditions (Supplementary Figs. 1 and 2), so it does appear to be playing a role in the cell.

These results provide evidence from multiple approaches supporting the characterization of a unique gene family in *Tetrahymena*, some of which may be lipases associated with lipid droplets. One function of lipid droplets is to store and release lipids as cellular energy. Consequently, lipid droplets often harbor an abundance of lipases that are responsible for the breakdown of TAG and mobilization of fatty acids based on cellular energy needs. Our previous work supports a model in which lipases are important during starvation, possibly to provide substrates for beta oxidation of stored TAGs for energy production during nutrient deprivation (Listenberger et al. 2025). In support of this model, most of the genes identified in this study show expression during starvation. Several of these genes also show a dramatic increase in expression during conjugation, a stage when cells undergo energetically costly events that require significant membrane remodeling (Nusblat et al. 2012; Cole et al. 2018).

## Supplementary Material

jkag117_Supplementary_Data

## Data Availability

Strains and plasmids are available upon request. Sequence data are available at the *Tetrahymena* Genome Database, NCBI GenBank, or the *Tetrahymena* Comparative Genome Database, and all accession numbers for sequences used in this study are listed in Supplementary Table 1. The authors affirm that all data necessary for confirming the conclusions of the article are present within the article, figures, and tables. Supplemental material available at G3 online.
