# Supplementary figures and images for: Bioinformatic, cell localization, and phylogenetic analyses reveal a novel family of putative lipases in Tetrahymena thermophila
Source: G3 (Bethesda). 2026 May 2;16(7):jkag117. doi: 10.1093/g3journal/jkag117 (PMC13334175; doi:10.1093/g3journal/jkag117)

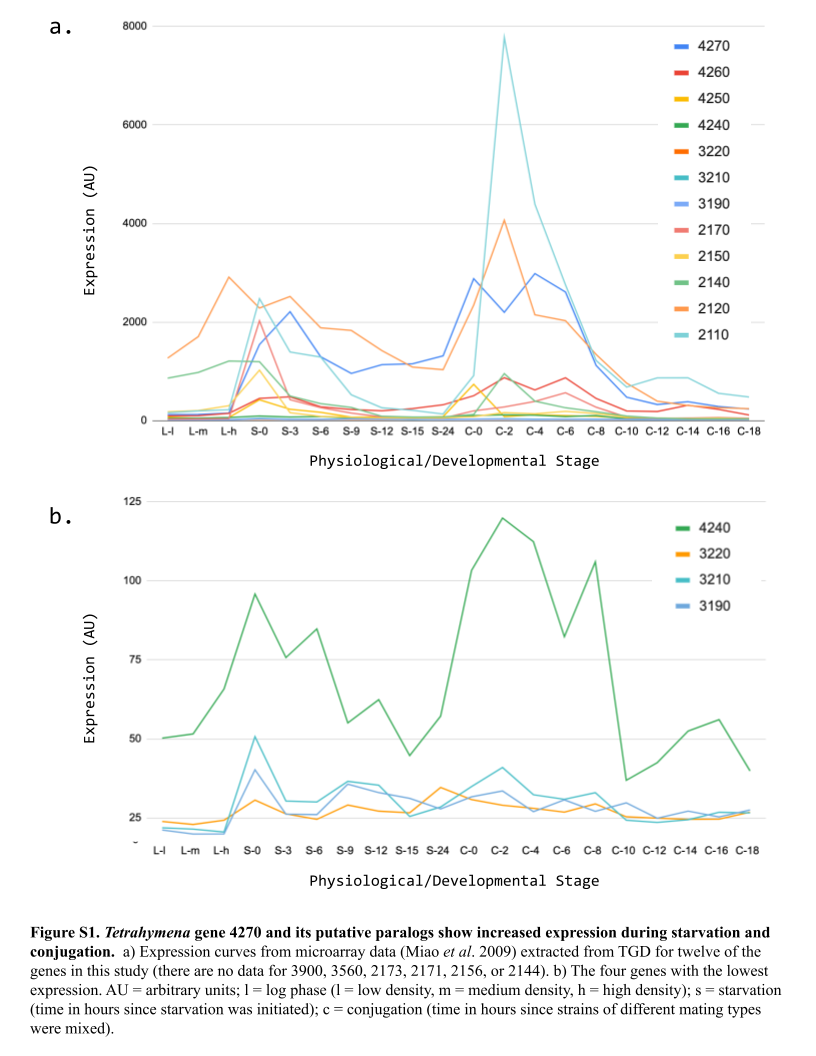

Supplement: jkag117_Supplementary_Data [file jkag117_supplementary_data.zip › Figure_S1_G3-2026-406813.png]

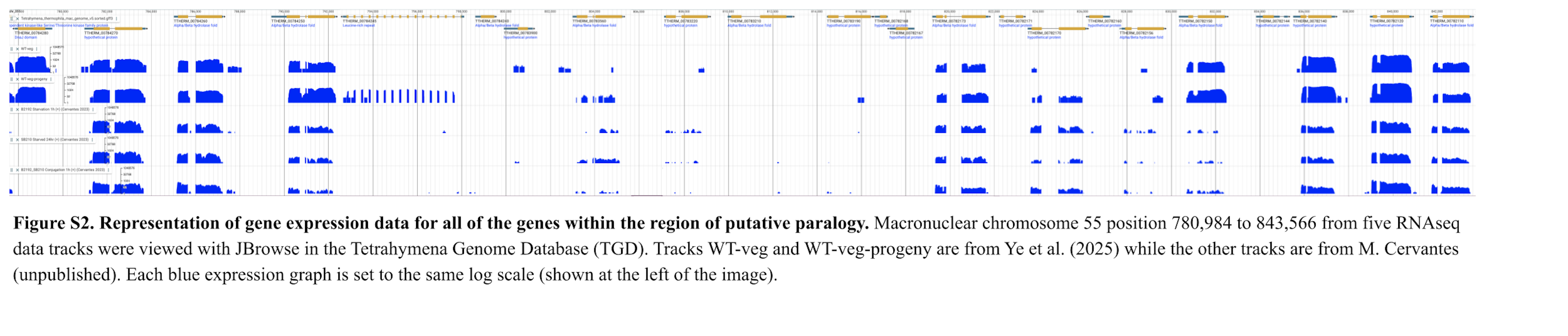

Supplement: jkag117_Supplementary_Data [file jkag117_supplementary_data.zip › Figure_S2_G3-2026-406813.png]
